# Steady-state mycophenolate mofetil pharmacokinetic parameters enable prediction of systemic lupus erythematosus clinical flares: an observational cohort study

**DOI:** 10.1186/ar3202

**Published:** 2010-12-22

**Authors:** Sarah Djabarouti, Dominique Breilh, Pierre Duffau, Estibaliz Lazaro, Carine Greib, Olivier Caubet, Marie-Claude Saux, Jean-Luc Pellegrin, Jean-François Viallard

**Affiliations:** 1 Laboratoire de Pharmacocinétique et Pharmacie Clinique EA2968, Université Victor Segalen Bordeaux 2, Hôpital Haut-Lévêque, CHU de Bordeaux, Avenue de Magellan, 33604 Pessac Cedex, France; 2 Service de Médecine Interne et des Maladies Infectieuses, Université Victor Segalen Bordeaux 2, Hôpital Haut-Lévêque, CHU de Bordeaux, Avenue de Magellan, 33604 Pessac Cedex, France

## Abstract

**Introduction:**

The aim of this study was to determine whether mycophenolate mofetil (MMF) pharmacokinetics (PK) under combined MMF and prednisone remission-maintenance therapy can predict systemic lupus erythematosus (SLE) clinical flares.

**Methods:**

At inclusion, steady-state PK parameters of the MMF active form, mycophenolic acid (MPA), and its glucuronide metabolite (MPAG) were determined for 25 stable SLE patients without renal manifestations. Disease activity was assessed during 6 months of follow-up. Potential relationships between those entry MMF-PK variables and clinical outcome were analyzed.

**Results:**

MMF controlled disease activity in 17 patients (successes) and failed to do so for 8 others (failures). For failures and successes, respectively, entry MPA areas under the time-concentration curve between 0 and 12 hours (AUC_0-12 h_) (medians: 37.7 vs 73.1 mg/h/L, *P *= 0.003) and MPA 12-hour trough concentrations (C_12 h_) (medians: 1.5 vs 3.7 mg/L, *P *= 0.008) were significantly lower, and inclusion MPAG/MPA C_12 h _ratios (medians: 18.7 vs 10.2, *P *= 0.02) were significantly higher. According to our receiver operating characteristics curve analysis, MPA C_12 h _was best able to discriminate a flare during follow-up (93% sensitivity, 85% specificity). A 3-mg/L cut-off had 92% negative-predictive value for developing a flare during follow-up.

**Conclusions:**

For our SLE patients without renal manifestations, clinical flares developing under maintenance therapy were associated with steady-state inclusion MPA C_12 h _< 3 mg/L.

## Introduction

Systemic lupus erythematosus (SLE) is a chronic remitting-and-relapsing disease that engenders considerable health-care costs for each patient and may require long-term treatment with immunosuppressive agents [1]. Mycophenolate mofetil (MMF), an immunosuppressant widely used to prevent solid organ transplant rejection [2], is being used increasingly to induce SLE remission of minor relapses and to maintain remission after induction therapy [1]. No recommendations for optimal MMF dose are available for these patients. The dose used to control SLE activity usually ranges from 1.5 to 3 g daily and is based largely on clinical experience, which is very limited. However, most patients experience periodic disease flares, so maintaining disease control remains a challenge. The lupus flare rate varies among studies, often because of the different populations studied [1].

In the transplant setting, the observed association between drug exposure and the risk of acute rejection has encouraged the routine determination of areas under the time-concentration curve between 0 and 12 hours (AUC_0-12 h_) of mycophenolic acid (MPA), the MMF active form [2]. MPA monitoring after MMF administration in kidney transplant recipients improved patient outcomes over currently recommended fixed-dose strategy [3], although controversy persists [4]. Conversely, for patients with SLE, very few data are available on MMF pharmacokinetic-pharmacodynamic (PK-PD) relationships: an association between disease activity and plasma MPA levels was recently demonstrated in patients with autoimmune diseases [5], as was a concentration-effect relationship between MPA exposure and SLE immunological activity [6].

In light of those findings, we postulated that inadequate immunosuppressive maintenance therapy for SLE reflected insufficient exposure to MPA. This study was conducted to evaluate whether initial steady-state MMF PK parameters in SLE patients without renal manifestations in remission could be predictive of clinical flares. Therefore, we determined those PK parameters for MPA and its main glucuronide metabolite (mycophenolic acid glucuronide, or MPAG) in SLE patients and then examined the parameters' respective predictive values for flares over the next 6 months.

## Materials and methods

### Patients

This single-center, observational study evaluated PK-PD relationships for unselected SLE patients with only extrarenal manifestations, which were defined according to the American College of Rheumatology. Patients were routinely followed between September 2005 and January 2008 in our hospital's internal medicine department. Patients gave written informed consent before being included. The study was approved by our local ethics committee (CHU de Bordeaux).

At the time of inclusion, all patients had completed induction therapy and were on a maintenance regimen comprised exclusively of MMF and low-dose prednisone. The main indications for starting MMF were remission maintenance after induction therapy with pulsed cyclophosphamide or treatment of minor disease relapses of hematological, cutaneous, articular, pulmonary, and/or cardiac SLE manifestations. Five patients who suffered minor relapses had received first-line therapy with hydroxychloroquine or azathioprine and were switched to MMF before inclusion.

Patients who had active renal involvement (proteinuria of greater than 0.5 g/24 hours with urinary casts or hematuria or both), renal impairment (glomerular filtration rate (GFR) calculated according to the Cockcroft-Gault formula of less than 60 mL/minute per 1.73 m^2^), and/or hepatic dysfunction were not included, nor were patients who had used cyclosporine, cholestyramine, magnesium- or aluminium-containing antacid, rifampicin, and/or antiviral within the 30 days preceding study entry.

Inclusion steady-state PK parameters were determined for all patients who demonstrated SLE regression or stabilization for at least 1 month. Included patients were those who had been taking a stable MMF dose (1 to 3 g/day) for at least 1 month with either declining oral prednisone (0.25 to 0.5 mg/kg per day) or prednisone maintenance (5 to 10 mg/day) therapy. MMF dose adaptations were initially made on the basis of tolerance and clinical response, and the inclusion MMF dose was maintained throughout follow-up. To avoid a potential influence of prednisone doses on clinical outcome, a standard predefined tapering regimen was applied to all patients: the prednisone dose had been stable for at least 15 days for all patients before inclusion and was maintained for 2 weeks thereafter and then tapered throughout follow-up (5 mg every 15 days with the objective of withdrawing prednisone for patients receiving 0.25 to 0.5 mg/kg per day or 1 mg every 15 days for patients under maintenance doses until discontinuation). Patients who discontinued MMF because of intolerance or non-compliance or both were excluded from the final PK-PD analysis.

### Pharmacokinetic analysis

For each patient, nine blood samples were collected in ethylenediaminetetraacetic acid-containing tubes before and 0.5, 1, 2, 3, 4, 6, 8, and 12 hours after MMF administration. Plasma MPA and MPAG concentrations were determined with chromatographic assays coupled with mass spectrometry [7]. A non-compartmental model with extravascular input for plasma data was used to estimate maximum MPA and MPAG concentrations (C_max_), times to maximum concentration (T_max_), 12-hour trough concentrations (C_12 h_), and AUC_0-12 h_, which was estimated with the logarithmic trapezoidal rule, and their MPAG/MPA AUC_0-12 h _and C_12 h _ratios were calculated.

### Follow-up and clinical outcomes

The primary outcome measure was the occurrence of clinical flares during the 6 months following entry PK determinations. At each subsequent monthly visit, all patients underwent a complete physical examination and laboratory testing. Serum albumin, aspartate aminotransferase, alanine aminotransferase, and *γ*-glutamyltransferase concentrations and GFR were determined, and corticosteroid dose was recorded. Biological analyses were also performed: complete blood cell count, urinalysis, C3 and C4 assays, antinuclear antibody test, and anti-double-stranded DNA.

Clinical outcome was assessed with the Systemic Lupus Erythematosus Disease Activity Index (SLEDAI) score. A flare was defined as an increase in SLEDAI score of at least 3 points since the previous examination [8]. Outcomes of patients whose disease remained clinically stable or regressed (SLEDAI score stabilization or improvement) during follow-up were classified as successes, whereas outcomes of patients whose disease was not controlled by the maintenance regimen and who experienced a flare were considered failures.

### Statistical analyses

Patient characteristics and PK data are expressed as median and interquartile range (IQR). Mann-Whitney *U *test or Fisher exact test (univariate analysis) was used to assess the relationship between each demographic, biological, or PK parameter and clinical outcome. Correlations were established with the Spearman correlation test. Data were analyzed with Statistica software (version 6.1; StatSoft, Créteil, France). Receiver operating characteristic (ROC) curves were analyzed to determine which parameters could best discriminate a clinical flare. Areas under the ROC curves (AUC ROCs) and their 95% confidence intervals (CIs) were calculated using the method of Hanley and McNeil. AUC ROCs of potentially predictive parameters were compared using Analyse-it software (Analyse-it Software, Ltd., Leeds, UK). The threshold value providing the best trade-off between sensitivity and specificity would be recommended as the discriminant cutoff. A *P *value of less than 0.05 defined statistical significance.

## Results

### Patient characteristics

The demographic and disease characteristics and inclusion PK parameters of the 26 Caucasian SLE patients without extrarenal manifestations are shown in Table 1. As indicated by the SLEDAI scores, SLE was clinically stable or had regressed for at least 1 month at entry, and all patients met PK steady-state conditions. Twenty-one patients ingested 2 g/day of MMF; daily intake was 1 or 3 g for 2 patients each, and 1 patient took 1.5 g/day. Co-medications were oral prednisone (range of 5 to 45 mg/day), calcitriol, and proton-pump inhibitors for all patients. The median MPA AUC_0-12 h _was 64.7 mg/hour per L, with high between-patient variability, as attested by a coefficient of variation (CV) of 44%.

**Table 1:** Inclusion characteristics (of the 26 systemic lupus erythematosus patients) and their ability to predict clinical outcome (univariate analysis)

Inclusion characteristic	Total *n *= 26	Successes *n *= 17	Failures *n *= 8	*P *value^a^
Demographic				
Females/Males, number	16/10	12/5	4/4	0.28^b^
Age, years	46 (35-61)^a^	46.5 (35-61)	46 (38-61)	0.9
Body weight, kg	60 (56-75)	60 (56-75)	62 (58-82.5)	0.62
Disease				
Duration before entry, months	11 (7-14)	11 (7-12)	10 (6-13)	0.30
SLEDAI score	0 (0-2)	0 (0-2)	0 (0-2)	0.20
C3, g/L	1 (0.6-1.1)	1 (0.9-1.1)	0.8 (0.56-0.96)	0.21
C4, g/L	0.2 (0.1-0.2)	0.2 (0.1-0.2)	0.2 (0.16-0.24)	0.78
Anti-double-stranded DNA, IU/mL	24 (6-51)	8 (1 -32)	38 (9 -55)	0.19
Biological				
GFR, mL/minute	95 (67-125)	95 (67-125)	93.5 (76-109)	0.64
Albumin, g/L	40 (39.8-43.8)	40 (37.9-43.8)	42.8 (40-46.9)	0.33
Aspartate aminotransferase, IU/L	23 (19-26)	23 (17.5-24.5)	28 (20-33)	0.26
Alanine aminotransferase, IU/L	22 (15-29)	19 (13-24.5)	25 (19-37)	0.21
*γ*-Glutamyltransferase, IU/L	24 (17-63)	23 (17-21)	42 (17-61)	0.92
Treatment				
MMF, g/day	2 (2-2)	2 (2-2)	2 (2-2)	0.9
Corticosteroids, mg/day	11 (7-35)	10 (5-20)	15 (7-45)	0.37
Months of MMF therapy	2 (1-3)	2 (1-3)	2 (1-3)	0.56
Months of corticosteroids	10 (6-13)	10 (5-15)	9 (5-14)	0.12
MPA pharmacokinetic parameters				
AUC_0-12 h_, mg/hour per L	64.7 (38.2-82)	73.1 (61.8-95)	37.7 (32-43.7)	0.003
C_max_, mg/L	16.1 (9.5-18.5)	16.3 (9.7-17.4)	13.3 (7-22.5)	0.69
T_max_, hours	1 (1-2)	1 (1-2)	1.1 (1-2)	0.82
C_12 h_, mg/L	2.4 (1.5-4.1)	3.7 (2.3-4.9)	1.5 (0.6-2.1)	0.008
MPAG pharmacokinetic parameters				
AUC_0-12 h_, mg/hour per L	775.3 (475-1,026)	791 (635-1,166)	678.8 (426-840.6)	0.22
T_max_, hours	2 (2-3)	2.3 (2-3)	1.3 (1-2)	0.01
C_12 h_, mg/L	32.1 (24.3-41.9)	34.7 (26.4-49.2)	29.8 (15.5-40)	0.26
MPAG/MPA AUC_0-12 h _ratio	11.5 (8.3-20.7)	10.9 (6.2-14.8)	18.7 (14.1-22.7)	0.07
MPAG/MPA C_12 h _ratio	11.5 (6.8-17.3)	10.2 (6.3-15)	18.7 (11.5-47.2)	0.02

Values are expressed as median (interquartile range) unless stated otherwise. ^a^Mann-Whitney *U *test; ^b^Fisher exact test. AUC_0-12 h_, area under the plasma concentration-versus-time curves for hours 0 to 12; C_12 h_, 12-hour trough concentration; C_max_, maximal concentration; GFR, glomerular filtration rate (estimated with the Cockcroft-Gault formula); MMF, mycophenolate mofetil; MPA, mycophenolic acid; MPAG, mycophenolic acid glucuronide; SLEDAI, Systemic Lupus Erythematosus Disease Activity Index; T_max_, time to maximal concentration.

No significant correlations were observed between MPA AUC_0-12 h _and serum albumin (*r *= -0.14, *P *= 0.2) or body weight (*r *= 0.22, *P *= 0.3). Plasma MPAG was a mean of 15-fold higher than MPA (median MPAG AUC_0-12 h _of 775.3 mg/hour per L), with wide between-subject variability of glucuronidation (CV = 43%). The MPAG/MPA AUC_0-12 h _and MPAG/MPA C_12 h _ratios were highly correlated (*r *= 0.60, *P *= 0.005). A positive correlation was observed between MMF dose and MPA AUC_0-12 h _(*r *= 0.58, *P *= 0.01) or MPA C_12 h _(*r *= 0.53, *P *= 0.02). No significant correlation could be established between the C_12 h _ratios and serum albumin (*r *= 0.22, *P *= 0.3), GFR (*r *= 0.10, *P *= 0.70), or prednisone dose (*r *= -0.16, *P *= 0.2). Lower C4 concentrations tended to be correlated with lower MPA C_12 h _(*r *= 0.44, *P *= 0.08).

### Relationships between the entry steady-state pharmacokinetic parameters and clinical outcomes

During follow-up, SLE remained stable or regressed in 17 patients and relapsed in 8. The median MMF duration before the flare was 5 (IQR of 4 to 6) months. The specific SLE manifestations were new arthritis and active glomerulonephritis in 3 patients, arthritis or myositis or both in 2, and vasculitis or cardiorespiratory and neurological symptoms in 1 patient, and cytopenia and myositis in 1 patient. One patient had MMF side effects (diarrhea, nausea, and abdominal pain) requiring its discontinuation and thus was excluded from the PK-PD analysis. The others tolerated it well.

**Figure 1:**
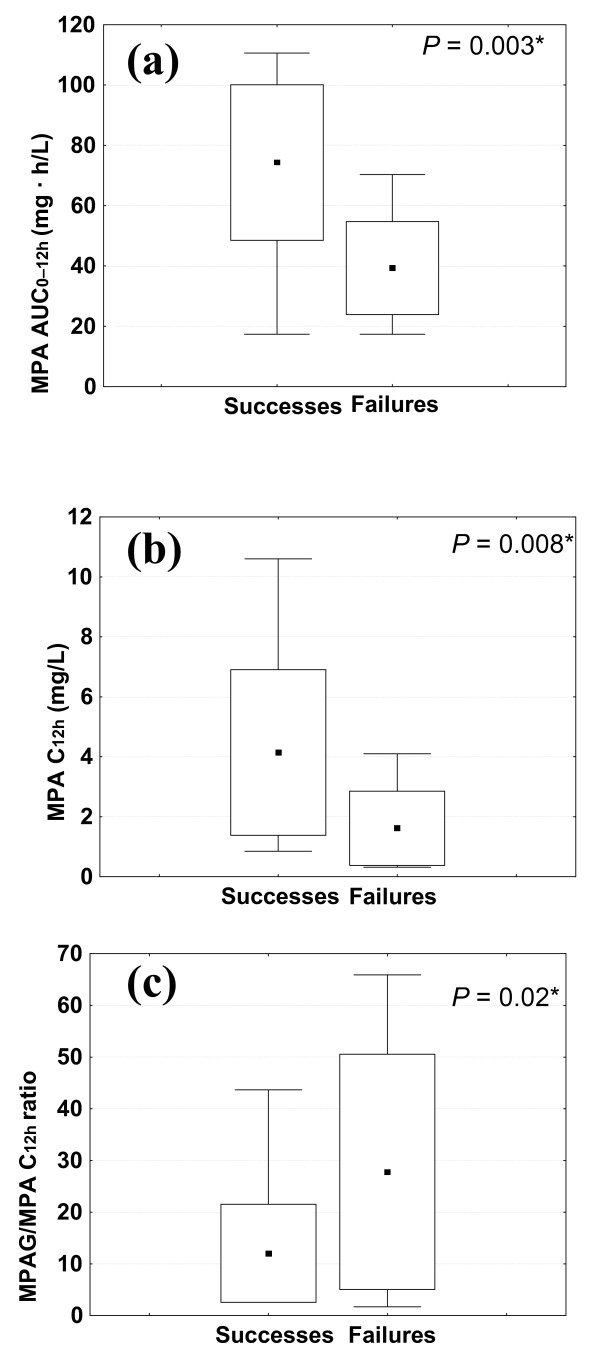
**Box plots at baseline for endpoint successes and failures**. **(a) **Area under the plasma concentration-versus-time curves between 0 and 12 hours of mycophenolic acid (MPA AUC_0-12 h_). **(b) **Twelve-hour trough concentrations of MPA (MPA C_12 h_). **(c) **Ratios of mycophenolic acid glucuronide (MPAG) to MPA C_12 h_. Black squares inside the boxes are means, the lower and upper box limits are standard deviations, and T bars correspond to the range. *Mann-Whitney *U *test.

The PK parameters were compared between the 17 successes and 8 failures (Table 1). Patients whose SLE remained clinically stable or regressed during the 6 months of follow-up had baseline MPA AUC_0-12 h _and MPA C_12 h _that were significantly higher than those of patients whose disease flared. The latter had significantly higher MPAG/MPA C_12 h _ratios (Figure 1a-c) and significantly lower MPAG T_max_. Successes and failures had comparable median inclusion prednisone doses (Table 1). Prednisone doses did not differ statistically between successes and failures at endpoint (respective medians of 5 (IQR of 5 to 5) mg versus 5.5 (IQR of 5.5 to 7.5) mg; *P *= 0.60).

### Receiver operating characteristic curve analysis

**Figure 2:**
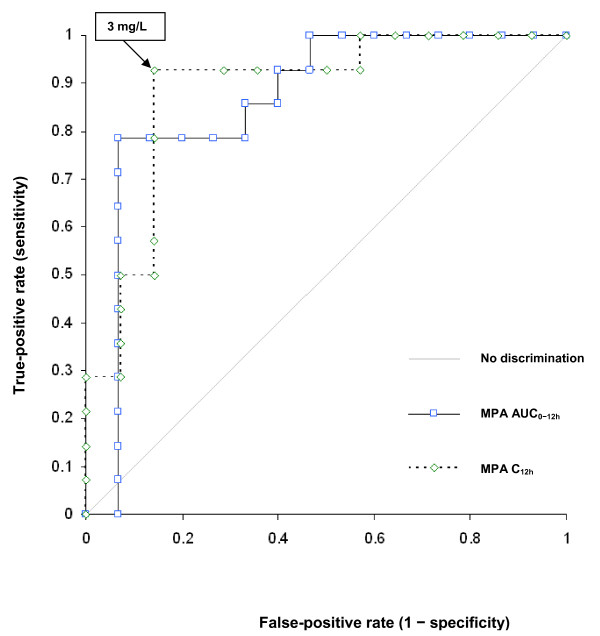
**Receiver operating characteristic curve estimates of area under the plasma concentration-versus-time curves between 0 and 12 hours of mycophenolic acid (MPA AUC_0-12 h_) and 12-hour trough concentration of MPA (MPA C_12 h_)**.

AUC ROCs were 0.86 (95% CI 0.71 to 1), 0.89 (95% CI 0.75 to 0.98), and 0.75 (95% CI 0.56 to 0.90), respectively, for MPA AUC_0-12 h_, MPA C_12 h_, and MPAG/MPA C_12 h _ratio. Comparison of AUC ROCs showed no significant difference between MPA C_12 h _and AUC_0-12 h _(*P *= 0.77). MPA AUC_0-12 h _and C_12 h _were strongly correlated (*r *= 70, *P *< 0.05), but the latter appeared to be better able to discriminate a clinical flare during follow-up, with 93% sensitivity and 85% specificity (Figure 2). A threshold of 3 mg/L had 92% negative predictive value for developing a flare during follow-up.

## Discussion

Although the majority of our patients received the standard MMF dose of 2 g/day, broad interpatient CVs for its PK parameters were observed. Moreover, 31% of our patients relapsed during the 6-month follow-up while on this recommended dose. Considering the attention focused on individualizing MMF therapy for transplantees [3], we postulated that SLE flares occurring under maintenance therapy reflected insufficient exposure to MPA, the MMF active form. Therefore, we examined the possible relationships between MMF PK parameters and SLE activity under maintenance therapy.

Significantly lower MPA AUC_0-12 h _and C_12 h _and higher MPAG/MPA C_12 h _ratios were observed at inclusion for patients deemed therapeutic failures during follow-up. Thus, SLE flares were associated with lower plasma MPA concentrations. Our univariate analyses did not find C3 and C4 levels or anti-double-stranded DNA antibody positivity to be predictive of such exacerbations. It is commonly accepted that SLE patients may suffer clinical flares without serological changes [9], but in our study, this circumstance could be explained by our small sample size. The only studied parameter differing significantly between clinical successes and failures was entry steady-state MPA exposure.

The ROC curve analysis showed MPA C_12 h _to be as effective a marker as AUC_0-12 h _to discriminate a clinical flare during follow-up. That observation contradicts findings in the transplantation setting, in which a poor correlation between MPA C_12 h _and AUC_0-12 h _was described [2], making the latter the marker of choice to prevent acute rejection.

According to our results, therapeutic drug monitoring based on the simple measurement of MPA C_12 h _enabled the prediction of SLE patients' clinical outcomes. Notably, our data are in agreement with those of two studies, one conducted on 20 patients with SLE [6] and the other on 39 patients with SLE or vasculitides [5], and confirmed the results reported in the latter. First, our study population was characterized by a mean ± standard deviation MPA AUC_0-12 h _of 64 ± 28 mg/hour per L, which is very close to their value of 66 ± 22 mg/hour per L [5]. Those authors also proposed a C_12 h _threshold of 3 mg/L for MPA monitoring. This threshold provided the best trade-off between sensitivity and specificity, with 92% negative predictive value in our study.

Unfortunately, our results cannot be compared with those obtained by others, mainly because our inclusion criteria were more restricted, as we enrolled only SLE patients without renal manifestations to ensure clinical homogeneity of the population. Because of the heterogeneous nature of clinical autoimmune disease manifestations, flares are often evaluated using an activity index. More than 20 different lupus activity scales have been used, and the Birmingham vasculitis activity score is used widely in therapeutic studies on systemic vasculitides. We thought that pooling systemic vasculitis and SLE patients would prevent a clear general definition of 'flare' and thereby introduce bias into the determination of efficacy thresholds.

We used one of the best characterized and most routinely used SLE scores and defined a flare as an increase in SLEDAI score of at least 3 points since the previous visit (1 month earlier). Only severe clinical exacerbations requiring therapeutic changes were considered whereas mild and moderate flares were excluded, and this choice might represent a limitation of our study. Another possible limitation is that, owing to the small sample, the relationship between the PK and MMF side effects could not be examined. However, 70% of our patients had an AUC_0-12 h _of greater than 60 mg/hour per L without any adverse events. Third, patients were evaluated for only 6 months, and according to Posalki and colleagues [10], although disease activity may regress during the first year of MMF treatment, this immunosuppressant fails to prevent extrarenal flares when continued beyond 2 years. Thus, it is likely that our flare rate would have been higher if follow-up had been longer. Finally, an important limitation is our study's lack of statistical power and the participation of only one center, perhaps explaining why no association was observed between clinical outcome and variables (age, sex, or biological markers) other than PK. Consequently, we were not able to identify confounding variables (for example, age, weight, steroid doses, or sex) in a multivariate analysis [11]. However, for several reasons, we think that prednisone withdrawal could have had only a minor impact on clinical response: low prednisone doses at inclusion (median of 11 mg), the standard tapering regimen applied to all patients, and comparable median entry and endpoint prednisone doses for successes and failures.

Despite these limitations, our results confirmed the large interpatient CV of the MMF PK parameters for SLE patients, as previously described for transplant recipients [2], showed that therapeutic drug monitoring under MMF steady-state conditions in SLE without renal manifestations predicted the clinical outcome under maintenance therapy, and demonstrated that such PK analyses should be based on MPA C_12 h_, as previously reported [5]. We concluded that the discriminant threshold values were highly dependent on the study population and the disease activity index chosen. Indeed, our PK results and proposed efficacy threshold are different from those observed in a cohort that included a majority of patients with lupus nephritis [12]. This discrepancy is logical given the predominant influence of renal function on MPAG and plasma MPA exposure [2].

Therapeutic failures might benefit from optimized dosing. In our opinion, 31% of our patients should have been prescribed daily MMF doses higher than the standard 2 g. Indeed, the impact of higher MMF doses merits further study. One possible explanation for failures on MMF is the increased clearance suggested by the higher metabolite levels, as the MPAG/MPA C_12 h _ratio was significantly higher in patients who developed flares. Low serum albumin level, renal dysfunction, and co-medications (corticosteroids) are the main factors usually blamed for raising metabolite concentrations [2]. As expected based on our inclusion criteria and sample size, we did not establish any significant correlation between MPAG/MPA C_12 h _and serum albumin, corticosteroid dose, or GFR. Conversely, the MPAG T_max _was significantly lower for patients failing on MMF, thereby raising a hypothetically increased glucuronidation ability in failures, which favors MPA metabolism and excretion. Glucuronidation might be under genetic control, and this needs to be confirmed in future clinical studies [13].

## Conclusions

For our SLE patients without renal manifestations, the occurrence of disease flares under MMF and prednisone maintenance therapy was significantly associated with lower MPA C_12 h_. We now recommend a target threshold of 3 mg/L for our patients. Further studies on larger populations are needed to confirm the ability of MPA PK parameters to predict clinical outcomes of SLE patients.

## Abbreviations

AUC ROC: area under the receiver operating characteristic curve; AUC_0-12 h_: area under the time-concentration curve between 0 and 12 hours; C_12 h_: 12-hour trough concentration; CI: confidence interval; CV: coefficient of variation; GFR: glomerular filtration rate; IQR: interquartile range; MMF: mycophenolate mofetil; MPA: mycophenolic acid; MPAG: mycophenolic acid glucuronide; PD: pharmacodynamic; PK: pharmacokinetic; ROC: receiver operating characteristic; SLE: systemic lupus erythematosus; SLEDAI: Systemic Lupus Erythematosus Disease Activity Index; T_max_: time to maximum concentration.

## Competing interests

The authors declare that they have no competing interests.

## Authors' contributions

SD, DB, and J-FV designed the study, performed experiments, collected data, and wrote the manuscript. PD, EL, CG, and OC followed the patients, collected clinical data, and helped to interpret data. M-CS and J-LP helped to perform experiments, interpret data, and write the manuscript. All authors read and approved the final manuscript.
